# Measurements of inter-cochlear level and phase differences of bone-conducted sound

**DOI:** 10.1121/1.4983471

**Published:** 2017-05-23

**Authors:** Robert W. J. Mcleod, John F. Culling

**Affiliations:** School of Psychology, Cardiff University, Tower Building, Park Place, Cardiff, CF10 3AT, United Kingdom

## Abstract

Bone-anchored hearing aids are a widely used method of treating conductive hearing loss, but the benefit of bilateral implantation is limited due to interaural cross-talk. The present study measured the phase and level of pure tones reaching each cochlea from a single, mastoid placed bone transducer on normal hearing participants. In principle, the technique could be used to implement a cross-talk cancellation system in those with bilateral bone conductors. The phase and level of probe tones over two insert earphones was adjusted until they canceled sound from a bone transducer (i.e., resulting in perceived silence). Testing was performed in 50-Hz steps between 0.25 and 8 kHz. Probe phase and level results were used to calculate inter-cochlear level and phase differences. The inter-cochlear phase differences of the bone-conducted sound were similar for all three participants showing a relatively linear increase between 4 and 8 kHz. The attenuation characteristics were highly variable over the frequency range as well as between participants. This variability was thought to be related to differences in skull dynamics across the ears. Repeated measurements of cancellation phase and level of the same frequency produced good consistency across sessions from the same participant.

## I. INTRODUCTION

**FIG. 1.**
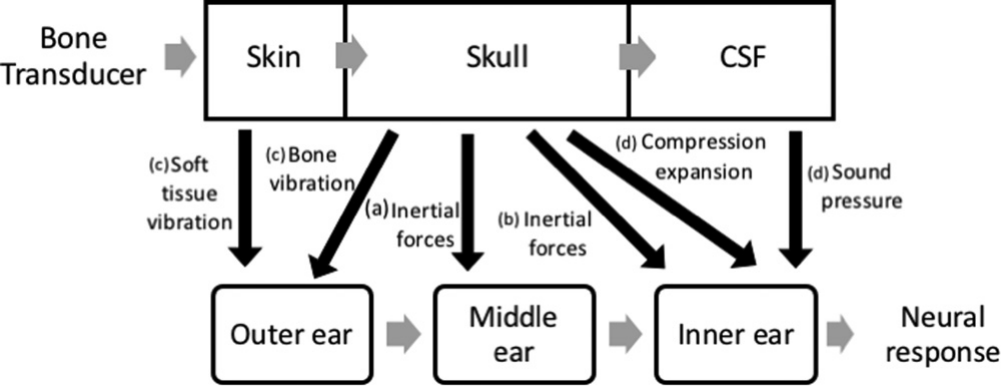
Overview of primary sound pathways via BC, adapted from Stenfelt (2011).

Bone-anchored hearing aids (BAHAs) generate vibrations, which travel through and around the cranium as well as the surrounding tissues (Stenfelt, 2012). For certain patients (particularly those with middle ear defects) this method of sound transfer can offer significant benefits over air conduction (AC) (Gripper *et al*., 2007). The exact mode by which bone conduction (BC) stimulation is audible was initially addressed by von Békésy (1932). He discovered that it was possible to cancel a 400-Hz tone transmitted via a bone transducer (BT) with binaural earphones after the AC sound phase and level was carefully altered. This led to the hypothesis that the initial sound-transfer paths were different for AC and BC but both culminate in stimulating the basilar membrane. Tonndorf (1966) later described several possible methods by which BC sound transfers to the basilar membrane. However, the relative contributions of these pathways have been disputed. Röösli *et al.* (2012) suggested that there were four major components which make up BC sound. These were (a) the inertial movement acting on the ossicles; (b) inertia of the inner ear fluid (Stenfelt and Goode, 2005a); (c) sound radiated into the external ear from the ear-canal wall and from skull and soft-tissue vibration (Stenfelt *et al.*, 2003; Stenfelt and Reinfeldt, 2007); (d) compression of the petrous bone and sound pressure transfer from the cerebro-spinal fluid (Puria and Rosowski, 2012). These are outlined in Fig. 1, adapted from Stenfelt (2011). Stenfelt later demonstrated that the most important mode of transmission was the effect of fluid inertia within the cochlea (Stenfelt *et al*., 2003; Stenfelt and Goode, 2005a).

Nolan and Lyon (1981) raised the question of whether sound transmitted by BC is received at the same intensity at the ipsilateral and contralateral cochleae and how this “transcranial attenuation” varied with frequency. Studies which have focused on the transcranial attenuation properties of the skull have used various methods with many investigating the difference in hearing threshold in a single-sided-deafness group when placing a BT on the mastoid bone of the hearing side and the deaf side (Nolan and Lyon, 1981; Stenfelt, 2012). Studies such as this have shown that there is considerable variability in attenuation from −23 to 37 dB across listeners and frequencies (Stenfelt, 2012). Stenfelt's (2012) comparison of attenuation in unilaterally deaf patients found that median attenuation was 3–5 dB for frequencies up to 0.5 kHz and 0 dB for frequencies between 0.5 and 1.8 kHz. Attenuation was much greater (10 dB) at higher frequencies (3–5 kHz). One limitation of these studies is that the measurement of transcranial attenuation assumes skull symmetry and therefore symmetrical attenuation. However, asymmetry in a complex shape such as the skull is well known from computer aided tomography (Wismer and O'Brien, 2010). Thus this research is mainly beneficial in investigating appropriate masking thresholds for BC sound rather than giving precise inter-cochlear level differences produced by a BT in a particular fixed location.

The present study made accurate psychoacoustic measurements of phase and level differences between the two cochleae from a single BT. It is our hypothesis that it may be possible to build on this methodology in order to accurately measure the phase and level of sound from patients with bilateral BAHAs. This data would allow the creation of a cross-talk cancellation system by which cross-talk from one BAHA can be canceled at the contralateral cochlea by signal of matched level and opposite phase from the other BAHA. Patients could then make use of interaural level difference cues, improving speech in noise thresholds. The current study only investigates a unilaterally placed BT, but an extension of the method would allow bilateral data collection to be able to collect the necessary phase and level data for a cross-talk cancellation system.

Measurements of phase and level have been made previously at the cochlea using similar techniques. However, they have been limited to relatively few frequencies (von Békésy, 1932; Zwislocki, 1953; Clavier *et al.*, 2010; Puria and Rosowski, 2012; Stenfelt, 2007). Studies which have investigated vibration and phase characteristics of the skull over a wide frequency range have used holographic interferometry (Dörheide and Hoyer, 1984; Hoyer and Dörheide, 1983) or laser-Doppler-vibrometer and accelerometer measurements (Stenfelt and Goode, 2005a). The present work is the first study to measure the level and phase of BC sound reaching both cochleae over a wide frequency range. There were two experiments. The first investigated a narrow frequency range on each experimental sitting in order to identify the “fine structure” of phase and level changes. It also allowed investigation of whether there were common patterns of cancellation level and phase between participants. The second experiment tested a wide frequency range on different occasions in each participant with the aim of elucidating the variation in results of cancellation phase and level between each sitting of the same participant. This informs how much variation in results may be due to a slight variation in BT placement position and coupling which is impossible to avoid without attaching the BT to an abutment. Both experiments utilised the same experimental methodology with the only variation being the frequencies which were tested.

## II. GENERAL METHODS

The following experimental methodology was approved by Cardiff University Psychology Department Ethics Committee.

### A. Apparatus

Matlab™ was used to generate tones at a sampling rate of 44.1 kHz over three channels with the ability to vary the level and phase of each channel independently. An 8-channel Echo Darla 24/96 DAC (Echo Audio, Santa Barbara, CA) passed signals through an 8-channel Behringer Powerplay Pro-8 Headphone Amplifier (Behrenger Music, Willich, Germany) to a pair of Etymotic ER2 insert earphones (Etymotic Research, Elk Grove Village, IL) and a B71W (Radioear, Eden Prairie, MN) BT for BC mastoid stimulation. ER2 earphones with ER1-14B eartips attached were used to present the AC sound. These earphones were selected over open-ear headphones to prevent contamination from air-borne sound produced by the BT from affecting the signal at the cochleae. To minimise differences between experimental sittings of the same participant and between different participants, specially adapted lens-less glasses were used. These were comprised of a highly flexible plastic attachment which held the B71W in position whilst causing very limited vibration of the glasses themselves. The glasses allowed lower variation in B71W placement, because the superior portions of both pinnae as well as the bridge of the nose were effectively used as a fixed-point reference tripod for the glasses to rest on. The attachment for the B71W onto the glasses aimed to position the BT 55 mm behind the opening of the external auditory canal. This is the recommended surgical placement position for a BAHA abutment (Battista and Ho, 2003). Testing was performed in a single-walled Industrial Acoustics Company sound attenuating booth within a sound treated room.

### B. Calibration

In order to stimulate the BT at an appropriate level one participant performed cancellation of BC sound using AC using the method described in Sec. II D at 1, 3, 5, and 7 kHz. Once cancellation was achieved the corresponding level of both the ER2s and the BT was varied by +5 and −5 dB. No noticeable change in cancellation quality was identified by the participant indicating linear output from both the BT and ER2s over this level range. The presentation level of the BT was then set to the initial presentation level for all participant testing.

### C. Exclusion and inclusion criteria

Participants with self-reported normal hearing and no previous history of otitis externa or ear surgery were included. Otological examination was performed on participants to check for earwax. Participants with the potential for wax impaction following deep insertion of ER1-14B eartips were excluded.

### D. Participants

Six participants were recruited. However, following ear examination, two were excluded and one further participant experienced temporary otalgia following one testing session and did not take part in further sessions. Therefore, three participants completed testing (age range 22–29 yrs).

### E. Testing procedure

**FIG. 2.**
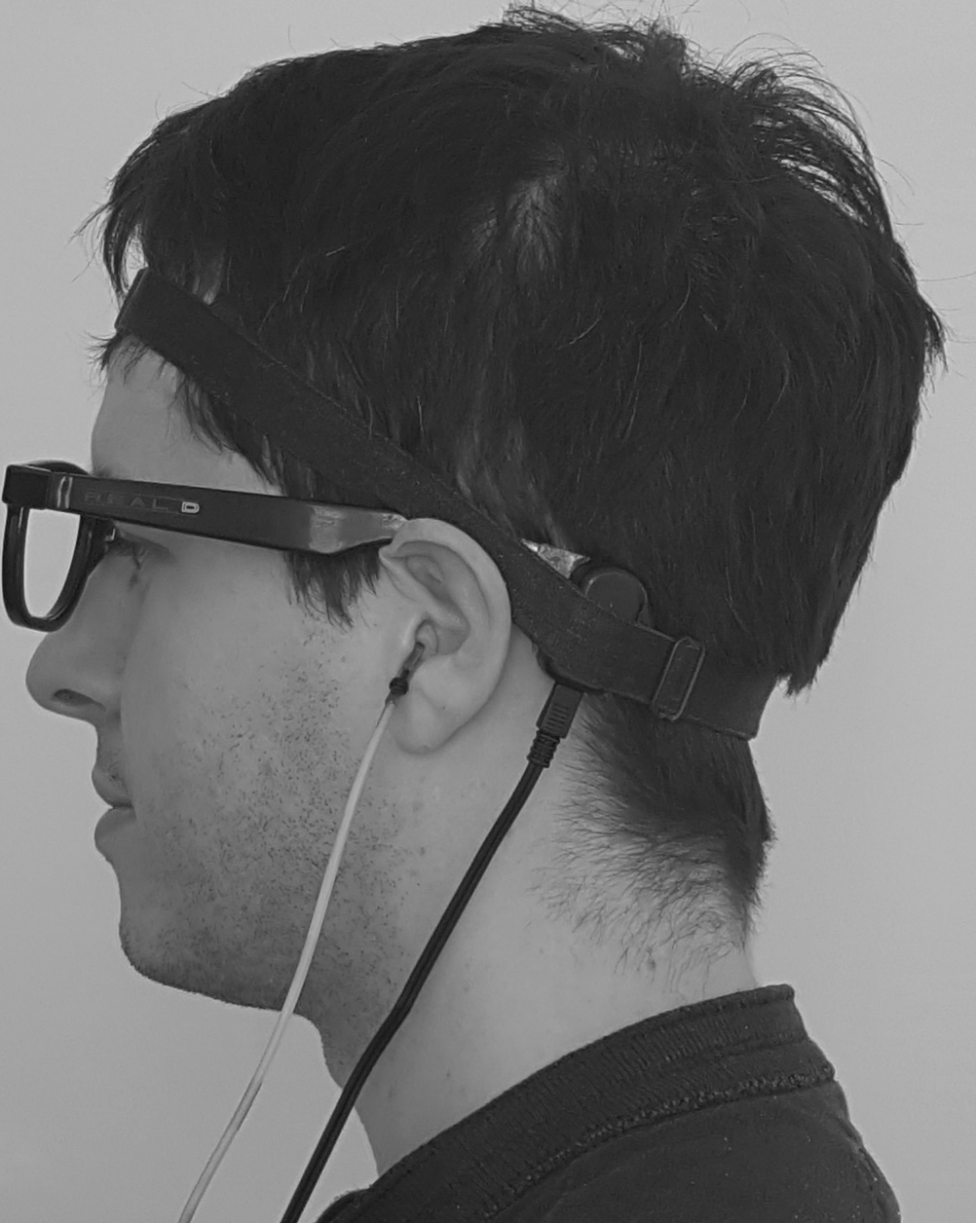
Image of BT placement via attachment to adapted lens-less glasses.

After deep insertion of eartips (approximately 22 mm in the ear canal), the BT was placed on the left mastoid and held in place by the adapted lens-less glasses (the left side will subsequently be referred to as the ipsilateral side and the right the contralateral side). Deep insertion was used to minimise the potential role of the occlusion effect; Stenfelt and Reinfeldt (2007) found that increased sound levels occurred only at frequencies below 400 Hz using this technique. A soft band was then placed over the participant's head and B71W BT in order to maintain a good acoustic coupling with the skull (see Fig. 2). The band was adjusted in order to achieve a static pressure of 2.5–3N as previously described by Reinfeldt *et al.* (2010). Participants performed eight separate 1-h testing sessions. Each session comprised a testing of several frequencies in a narrowband range (different on each session) and testing over a wide frequency range (repeated on each session). The testing of a high density of frequencies over a narrow band was designed to identify the “microstructural” changes in level and phase over this band. For clarity we have called this experiment 1. Retesting the same frequencies on each sitting revealed the variations caused by replacement of the BT over a number of experimental sittings. This will be referred to as experiment 2.

**FIG. 3.**
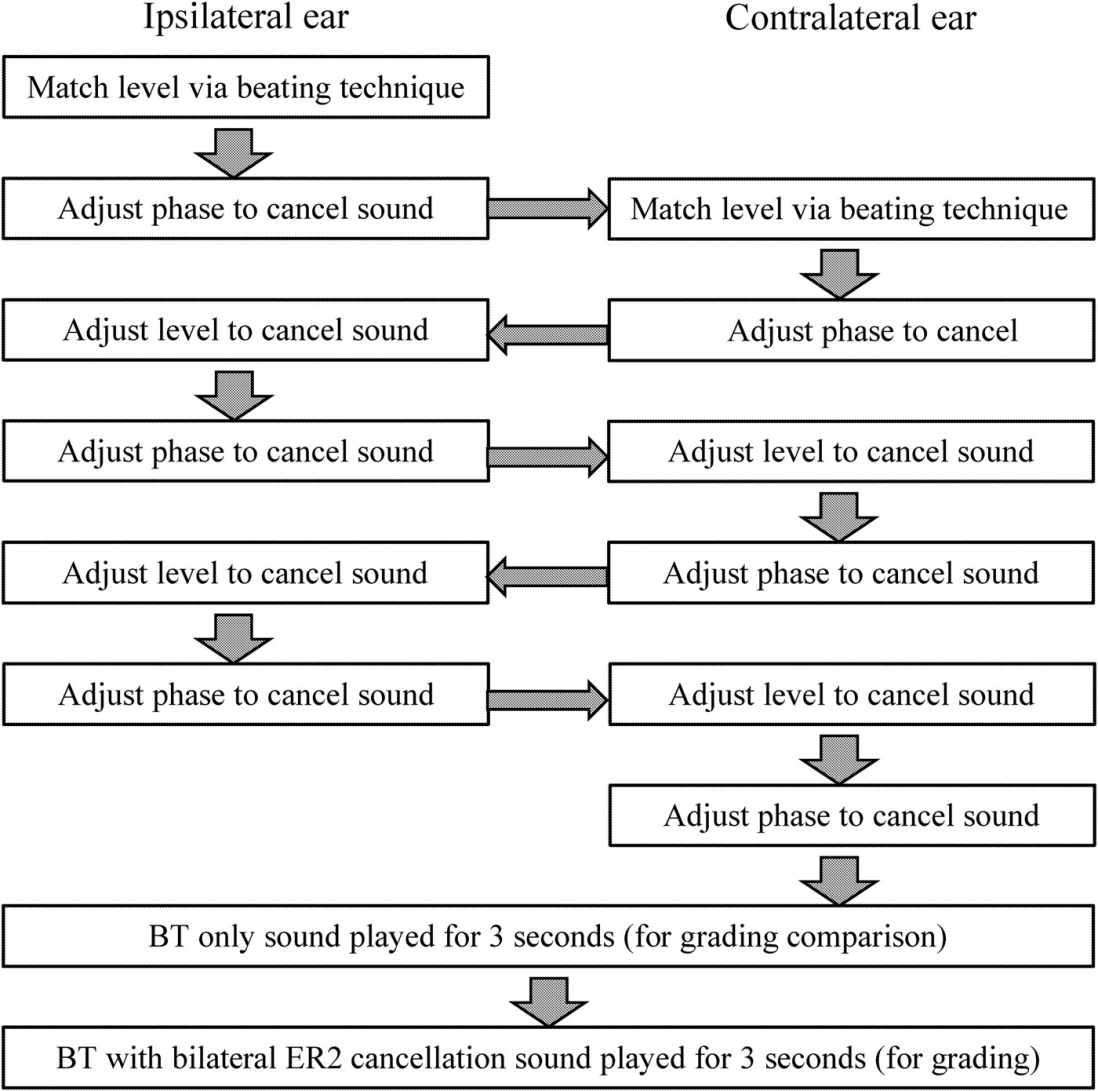
Illustration of the psychophysical procedure for cancelling a bone-conducted sound at both ears and providing an effectiveness rating.

Figure 3 shows the testing procedure undertaken at each frequency. During each test, a single target tone was presented via the BT at a fixed level and a 1-Hz higher tone presented via the ipsilateral ear over the ER2 earphones. No contralateral masking was used throughout the procedure. The participant was asked to vary the level of the ER2-presented tone in order to maximise the perceived beating effect as the two signals constructively and destructively interfered. Beating is known to be maximal when the levels of the signals at the basilar membrane are equal (Wever and Lawrence, 1954). Level changes were made using the scroller on a computer mouse. Each step of the scroller changed the level by 0.2 dB. Once the participant had selected an appropriate level, the same levels were presented again but using the same frequency in both the left ER2 earphone and the BT. Since the level of the AC and BC sound should be matched at the ipsilateral basilar membrane, the participant could then be asked to change the phase of the ER2-presented tone so as to minimise the perceived sound in the left ear. This procedure was intended to determine the phase at which destructive interference would occur and was again achieved using the mouse scroller, with each step of the scroller changing the phase by 2°. To cancel the signal going to the contralateral ear, the same processes of level adjustment followed by phase change were repeated on the right ER2 while the cancellation signal was simultaneously played on the left ER2. Participants were then asked to perform two further iterations of changing the level and phase in both ears in order to minimise the sound perceived at each ear. This procedure is illustrated in Fig. 3.

Following the adjustments to the phase and level of both ears, the initial un-canceled signal from the BT was presented for 3 s. This was then followed by the cancellation signal from the ER2 earphones with the BT signal for 3 s. Using Table I the participants were then asked to rate from 1 to 5 how well they had achieved cancellation (i.e., the reduction in loudness achieved). The purpose for this grading system was twofold. First, it indicated the relative difficulty of achieving cancellation at different frequencies. Second it allowed results in which the participant had not achieved good cancellation to be identified. Phase and level results from tests where grades of 1 were recorded were excluded from final analysis.

**TABLE I.** Grading system post attempted cancellation.

Grade	Description
1	As loud as start of task
2	Slightly quieter than BT alone
3	Much quieter than BT alone
4	Only slightly audible
5	Total cancellation (nothing audible)

In both experiments the order of frequency presentation was randomised to minimise practise effects. In experiment 1 this was achieved by randomising the sequence of the frequency bands tested as well as the order of increments (up or down) during each testing session. In experiment 2 the sequence of frequencies attempted were randomised. During each experimental session the BT position was not adjusted.

### F. Experiment 1

Participants performed testing every 50 Hz between 0.25 and 8 kHz with no frequency presented more than once. Testing was split into 8 sessions, which focused around a 1-kHz frequency band (for example, 3.05–4 kHz in 50-Hz increments).

The phase result of the lowest test frequency (250 Hz) was used as the anchor point from which all other phase results were unwrapped. Unwrapping was performed following completion of the eight testing sessions with phase results of the ipsilateral and contralateral sides relative to the BT. At the start of each experimental sitting there was no difference in computer generated tones delivered to the BT and ER2. In order to unwrap the data, phase results from the highest frequency attempted in one experimental sitting (e.g., 2 kHz from a 1.05–2 kHz) was compared to the lowest attempted on another experimental sitting (e.g., 2.05 kHz from a 2.05–3 kHz). For example, a cancellation phase of 355° at 2 kHz and a 5° phase for 2.05 kHz would result in the addition of 360° to all results between the 2.05 and 3 kHz testing session so that the unwrapped 2.05 kHz phase was 365°.

Calculation of the inter-cochlear phase and level was performed by subtracting the ipsilateral and contralateral unwrapped phases and levels. A positive value indicating the ipsilateral level was greater than the contralateral. We compared it to physical measurements from Stenfelt and Goode (2005a). Stenfelt and Goode employed accelerometers, which were attached close to the cochlea in severed cadaver heads. Several accelerometer positions were examined; however, the third occipital position was thought to be the closest to the recommended abutment placement position. Thus the mean accelerometer measurements placed in the third occipital position were used from six cadaver heads as a comparator. Stenfelt and Goode's data related to phase and level of vibrations in all three planes. However, the relative contribution to hearing of each plane of transmission are not known, therefore the dominant plane of transmission was used as a comparator. This was referred to by Stenfelt and Goode as the *x* axis, where vibrations were parallel to the sagittal plane. The difference between the phase and level measurements from the ipsilateral and contralateral *x* axis accelerometers in the third occipital plane were then used to estimate the inter-cochlear phase and level, as measured from the accelerometer position.

### G. Experiment 2

Experiment 2 utilised the same testing procedure as already outlined in Fig. 3. Participants performed 8 testing sessions of the same 8 frequencies (every 1 kHz between 1 and 8 kHz). The primary purpose of this experiment was to investigate the effect of small placement differences and coupling between the BT and skull on phase and level results needed for cancellation. In order to achieve this, the mean and standard deviation for each test frequency were calculated for the ipsilateral and contralateral phase and level.

## III. RESULTS

### A. Experiment 1 data exclusion

Participant 1 had 3 grading scores of 1. Participant 2 had 5 grading scores of 1 and participant 3 had 6 grading scores of 1. The raw ipsilateral and contralateral phase and level results related to these scores were excluded from further analysis. The majority of the results from these scores did not align with phase and level results for closely related frequency results and therefore this method was deemed appropriate for excluding outlier results.

### B. Experiment 1 phase data

**FIG. 4.**
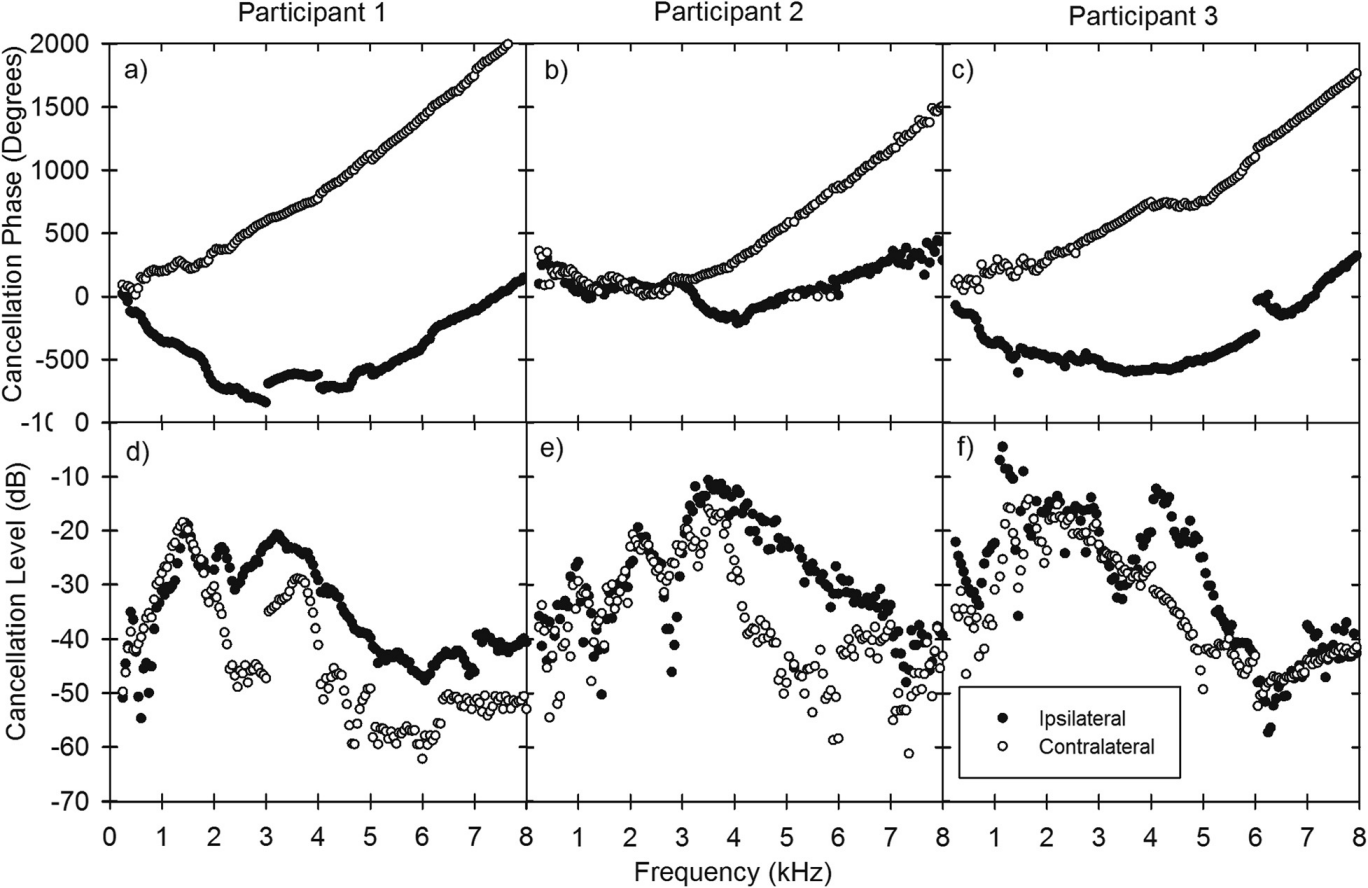
The raw cancellation phase (unwrapped) and level results from ER2 earphones needed at different frequencies to cancel a single B71W BT tone in three participants.

Figures 4(a)–4(c) show the raw unwrapped phase data for the ipsilateral and contralateral ER2 for the three participants. The data would include the effects of the transducers and their coupling to the head or ear, so the absolute values are not meaningful. However, it is clear from the results that, within each testing session, trends in phase seem to be very consistent, while between sessions there can be discontinuity in phase (clearest in participant 1 in the 3–4 kHz testing session). This shows that although using lens-less glasses as a method for B71W positioning provides considerable reliability, it has not completely resolved the problem of phase consistency between testing sessions.

### C. Experiment 1 level data

Figures 4(d)–4(f) show the raw ipsilateral and contralateral levels for cancellation using ER2s. The reference scale level is arbitrary but instead is of use to compare the relative levels needed for cancellation at each cochlea as well as be able to identify changes over frequency. As was evident in the phase results there are several discontinuities in level at the intersection of testing sessions. These are most clear in participant 1 in the contralateral ER2 at 3 kHz and participant 3 in the ipsilateral ER2 at 1 kHz.

### D. Inter-cochlear phase and level differences

**FIG. 5.**
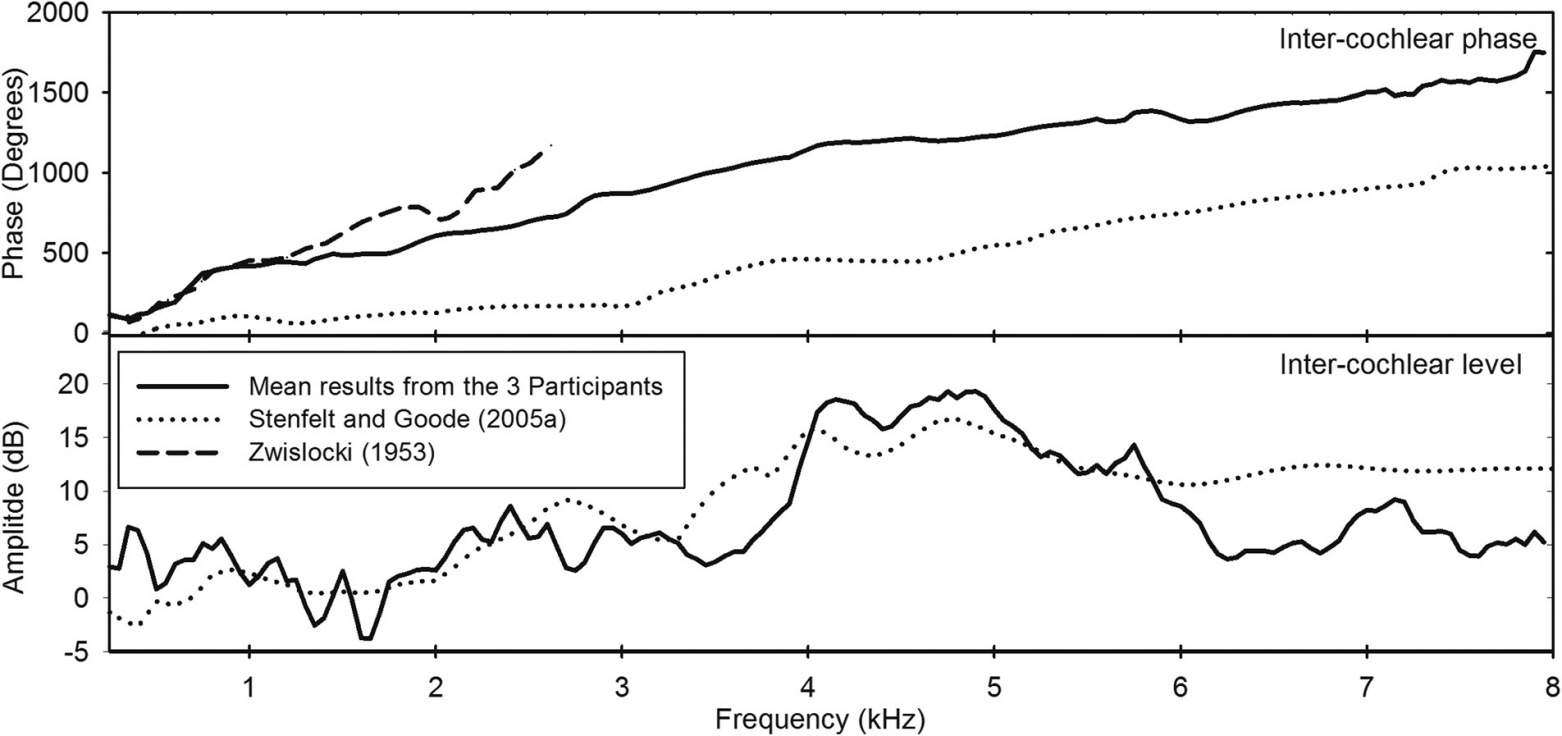
Mean inter-cochlear phase and inter-cochlear level data (Zwislocki, 1953) phase data (using a loud tone at one ear rather than a BT), and Stenfelt and Goode (2005a) inter-cochlear phase and inter-cochlear level difference (derived from accelerometer measurements of cadaver heads).

Figure 5 shows the mean inter-cochlear phases and levels calculated from the three participants using data from experiment 1. Inter-cochlear phase increases relatively rapidly at frequencies below 4 kHz before a gradual linear increase at higher frequencies.

The large discontinuities in level (seen between experimental sessions) that are apparent in Fig. 4 are no longer visible, indicating that these differences are primarily due to coupling variability which does not affect inter-cochlear measurements. Since cancellation thresholds are equally impacted on both sides, the effects of the changes in coupling, etc., are removed when the raw data at one ear is subtracted from that of the other to give the inter-cochlear differences.

Patterns in the inter-cochlear level were not as consistent across participants as those seen in the inter-cochlear phase. All participants had results with highly negative inter-cochlear level differences, although these occurred at different frequencies. Negative inter-cochlear level differences indicate that a greater level is needed for cancellation at the ear contralateral to the BT when compared to the ipsilateral ear. Participant 2 showed the greatest negative inter-cochlear level differences (−16.2) at 2.8 kHz, this was primarily due to a large reduction in the ipsilateral cancellation level.

For each participant, there were large drops (>10 dB) in the level needed to cancel BC sound over a relatively narrow frequency range (0.5 kHz). In participant 1, this was most marked at the contralateral ER2 at frequencies of 2, 4, and 4.5 kHz. In participants 2 and 3, the ipsilateral ER2 showed the most prominent acute reductions in cancellation level. These were identified at 1.5 and 2.8 kHz in participant 2 and 3.3 and 6.3 kHz in participant 3.

### E. Experiment 2 across-sitting variability

**FIG. 6.**
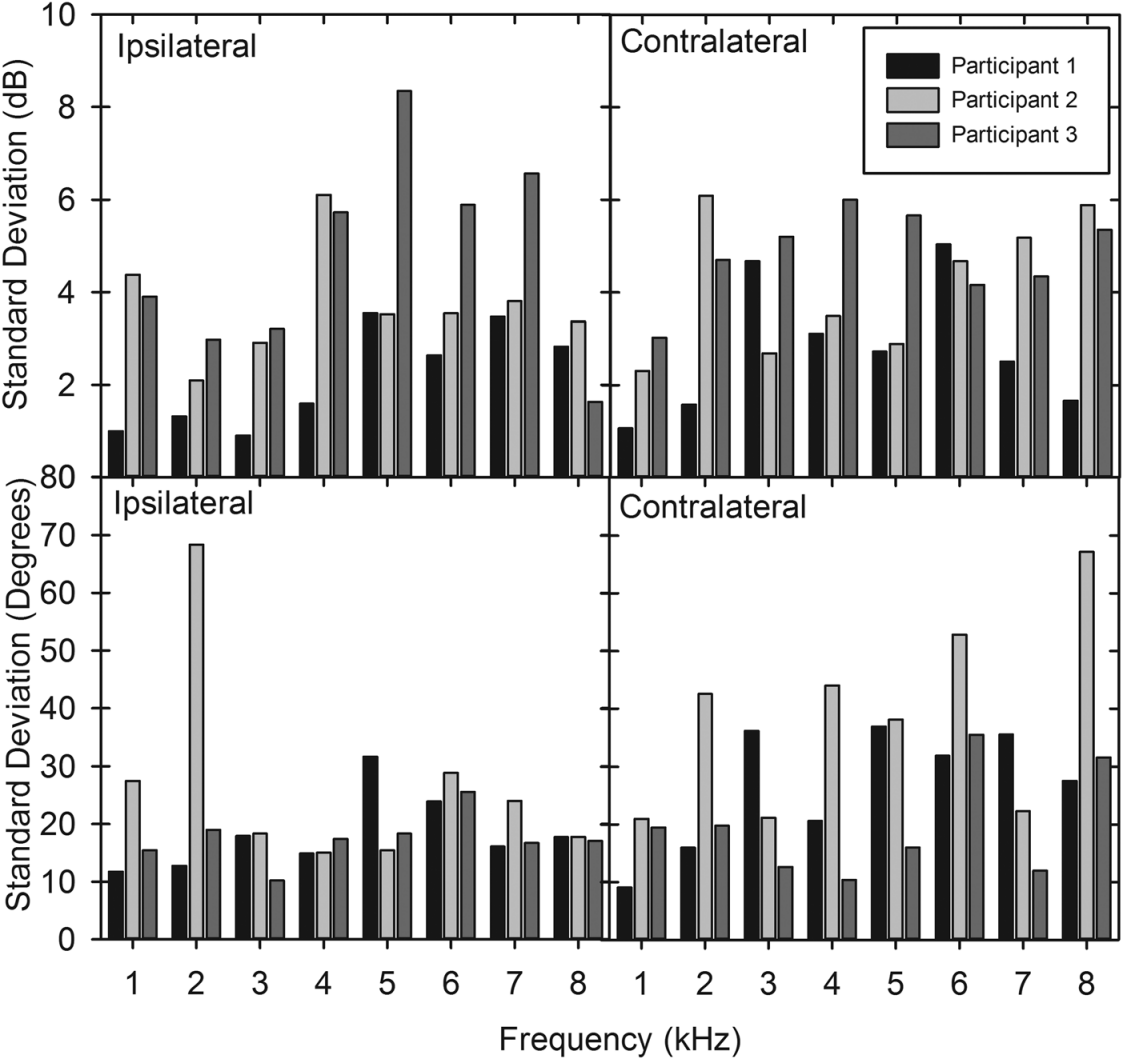
The standard deviation of the inter-session cancellation level and phase for each of the three participants. Results from eight different testing sessions.

Figure 6 shows the standard deviation of the ipsilateral and contralateral level and phase results for each participant (*n* = 8 for each participant). A paired student *t*-test found no significant difference in the standard deviation of level needed for cancellation when comparing the ipsilateral and contralateral ER2s (*t* = −0.73, *p* = 0.48). However, the standard deviation of the cancellation phase was greater in the contralateral ER2 when compared to the ipsilateral (*t* = −2.44, *p* = 0.02). The highest frequencies had the greatest standard deviation at the contralateral ER2.

Paired *t*-tests were also performed to investigate if there were consistent differences in level between the ipsilateral and contralateral sides. In one instance, the contralateral level was found to be significantly greater than the ipsilateral side. This was identified at 1 kHz in participant 1 where the mean difference between the ipsilateral and contralateral side was 3.8 dB (*t* = −2.97, *p* < 0.01). At all other repeated frequencies in all participants the mean level needed for cancellation was higher at the ipsilateral side. However, this difference was not significant in participant 1 at 2, 3, and 8 kHz and in participant 3 at 2, 3, 6, 7, and 8 kHz. Participant 2 showed a significantly higher level needed for cancellation across all frequencies at the ipsilateral side (*p* < 0.01).

### F. Experiment 2 grade data

**FIG. 7.**
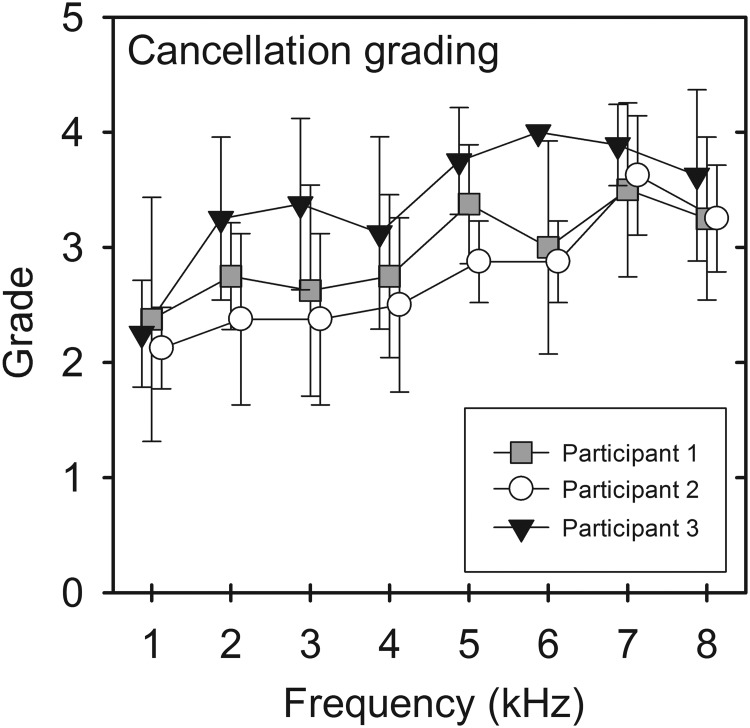
The mean cancellation grade (*n* = 8) for each of the participants. Error bars show one standard deviation.

Figure 7 shows the mean grade results of the eight testing sessions. Grading scores are lowest in all of the participants at the lowest frequency of 1 kHz. There was a general increase in grades between 1 and 7 kHz reflecting perceived better cancellation at higher frequencies.

## IV. DISCUSSION

### A. Cancellation phase change with frequency

There were consistent findings in all three participants for both ipsilateral and contralateral phase results. Linear increase in phase of the ipsilateral ER2 was identified at all but the lowest frequencies (<1 kHz). The ipsilateral phase reduced with increasing frequency up to approximately 3 kHz before a relatively linear increase. The linear incline in the ipsilateral phase was not as great as for the contralateral cancellation phase.

All three participants had a greater cumulative inter-cochlear phase when comparing findings with Stenfelt and Goode's (2005a) accelerometer measurements from cadaver heads. However, phase progression (as defined as the change in phase over a frequency range) was similar at frequencies above 4 kHz where wave motion predominates. Our results identified a greater accumulation of phase cycles than Stenfelt and Goode (2005a). It is unclear why there were differences between the two experiments, although one possible explanation for the difference relates to intracranial pressure (ICP). This is defined as the mean arterial pressure minus the cerebral perfusion pressure. Normal values are between 5 and 15 mmHg (Dunn, 2002). von Békésy (1932) described how a canceled BT signal is audible during coughing. Additionally, Voss *et al.* (2010) demonstrated significant changes in distortion product otoacoustic emission magnitudes when the participants' posture was altered. Since ICP changes during coughing and alterations in posture it may indicate that ICP has a significant influence on BC sound. Stenfelt and Goode's laser Doppler measurements were performed on cadaver heads within which the ICP would likely be 0 mmHg. Since Stenfelt has previously shown that fluid inertia within the cochlea is the main mode of sound transmission in bone-conducted sound this could have implications for the Doppler results. Further research is needed to investigate the role of ICP in cancellation results. Manipulation of the ICP would allow the investigation of how ICP changes the phase at different frequencies. This may then explain differences between Stenfelt's and Goode's phase findings and our own.

### B. Cancellation level changes with frequency

Inter-cochlear level differences were less consistent across participants when compared to phase. This fits well with previous studies (Nolan and Lyon, 1981; Stenfelt, 2012; Stenfelt and Goode, 2005b). Further investigation of inter-cochlear level differences (shown in Fig. 5) found that in the majority of cases participants had a positive inter-cochlear level difference indicating a greater perceived level at the ipsilateral cochlea. This is to be expected as dampening will be experienced over a greater distance in the contralateral cochlea when compared to the ipsilateral resulting in greater energy dissipation (Stenfelt, 2012). However, at some frequencies there were large negative inter-cochlear level differences. These strong lateralisation effects have been reported previously and are thought to be due to resonance and anti-resonance (Håkansson *et al*., 1996; Stenfelt *et al*., 2000; Tonndorf and Jahn, 1981). Anti-resonance can occur when sound pathways take different routes, which causes them to destructively interfere at or before the basilar membrane. Previous studies have concluded that the anti-resonance frequencies, which usually occur at the ipsilateral ear, may explain the large differences in the literature on transcranial attenuation properties (Eeg-Olofsson *et al*., 2011; Stenfelt and Goode, 2005a; Stenfelt, 2012; Stenfelt *et al*., 2000). Further research is needed to investigate whether the large drops in the cancellation level identified in the raw data are due to the intrinsic properties of the ossicles, cochlea, or temporal bone, or if it is also dependent on BT position or the occlusion effect.

In addition to the level changes identified in raw measurements it is also possible that variations in conductive hearing efficiency between the ears could contribute to inter-cochlear level differences. However, we feel that this is unlikely since participants were young, at low risk for hearing loss, and had normal tympanic membranes. Future studies using a similar technique but comprised of participants with ear pathology may identify a greater variation or difference in inter-cochlear level. Although for the inter-cochlear values to be used in a cross-talk cancellation system the accuracy of measurement is of greater importance than the difference itself.

Our measurements corresponded well with Stenfelt (2012) transcranial attenuation data, with low or negative (indicating a higher level on the contralateral side) inter-cochlear level differences at low frequencies. In both studies the differences in level increased to around 10–15 dB from 4 kHz before a small reduction at frequencies above 6 kHz.

### C. Test retest standard deviation

There was no difference in the standard deviation between the ipsilateral and contralateral cancellation levels. This may indicate that small variations in coupling and position which occur on repeated placement of the BT affect both sides equally and that the task is of equal difficulty irrespective of the side of the BT.

The standard deviation of the phase for the contralateral ear was found to be significantly greater than the ipsilateral ear, indicating variations in BT placement are more critical to the phase at the contralateral cochlea. Additionally, the standard deviation at the contralateral ear increased with frequency. This indicates that small variations in placement positions can make large differences in cumulative phase in the contralateral cochlea when compared to the ipsilateral cochlea and that these differences increase with frequency. This could be due to several different vibrational pathways interacting to stimulate the contralateral cochlea, whilst the ipsilateral cochlea may be more likely to have a “dominant” vibrational pathway and is thus less likely to be affected by a small change in position. It was unclear why participant 2 was found to have a large increase in standard deviation at the 2 kHz level. It may be that the participant was not achieving good cancellation at this frequency on two separate occasions but maintained a good grading so that the results were not excluded from analysis. An alternative explanation is that at 2 kHz there are two transmission pathways which are of similar level and that they are interacting to cause large changes in phase despite only small changes in placement position.

### D. Grading

All participants reported that cancellation was “best” achieved between 4 and 7 kHz. The most difficult frequencies to cancel were the lowest frequencies (under 1 kHz). All participants had a similar pattern of grading with a steady increase in grade from 1 kHz up to 7 kHz, which corresponds with greater perceived cancellation at the cochlea before a slight fall at 8 kHz.

During collection of grading data, the degree of lateralisation (indicating whether only one side was poorly canceled compared to the other) was not considered. Thus a poor cancellation grading at one frequency would result in both the ipsilateral and contralateral phase and level results being excluded. This method was used as it was felt that if the ipsilateral cochlea cancellation was performed poorly then the contralateral cochlea cancellation would also likely be inaccurate.

The lowest mean grading was identified at 1 kHz; this was likely due to the greater influence of lateralisation produced by inter-cochlear phase differences (Clavier *et al*., 2010; Rowan and Gray, 2008). This makes the initial task of accurately detecting maximal beating more challenging, impacting the accuracy of the rest of the task. Additionally the skull is thought to act as a mass spring between 0.3 and 1 kHz (Stenfelt, 2011). At these low frequencies the two temporal bones will be vibrating approximately out of phase. It could therefore be presumed that the psychoacoustic effect of this would be that participants experienced a beat at each ear at opposite points in the phase cycle. If this was indeed occurring, then it would make the level adjustment even more challenging.

### E. Future research

This study has shown consistent, progressive patterns in the inter-cochlear phase and level of BC sound within the same participant. Future studies employing a similar technique should find it possible to significantly speed up the testing procedure by using level and phase results for one frequency to predict the level and phase of similar frequencies. By doing this, a number of experimental steps can be avoided. This might allow the same spectral sampling to be achieved over a single 1-h testing session as opposed to eight 1-h testing sessions. The speed at which such data can be collected could be critical as it could make potential applications for inter-cochlear phase and level values more appealing. Such values could be key in the creation of cross-talk cancellation systems for bilateral BAHA users whereby two BAHAs are employed with the sound from one BAHA reaching the contralateral cochlea canceled by appropriately filtered sound delivered by the ipsilateral BAHA. In order to achieve this, the level and phase of sound from each BAHA needs to be known accurately at each frequency, as well as the inter-cochlear differences. This method could allow the creation of such a system (Liao, 2010). In order to investigate if this is possible we plan to perform studies to investigate if the use of this single BT method can be used to accurately predict the inter-cochlear phase and level produced by two BTs, where one is used to cancel the signal from the other.

A potential problem for the development of a cross-cancellation system is that the transmission of BC sound to each cochlea may depend on such factors as jaw position, or posture. Further studies are needed to evaluate the degree of variability in the results that these factors would introduce.

Other potential uses for BC cancellation include the creation of hearing protection systems where despite protection of AC sound, the BC pathways are still high enough to cause sensorineural hearing loss, such as on an aircraft carrier flight deck (Puria and Rosowski, 2012). The limit of hearing protection via conventional means is commonly known as the BC threshold (Reinfeldt *et al*., 2007). In theory knowledge of the phase and level of sound reaching each cochleae could be used to create an out of phase sound of the same level to cancel the BC sound, thus overcoming the BC threshold barrier which currently limits hearing protection devices.

## V. CONCLUSIONS

Using a single BT and ER2 headphones, we have demonstrated that it is possible to achieve repeatable phase results in both the ipsilateral and contralateral ears in binaurally hearing participants on multiple separate testing sessions over a large frequency range from 0.25–8 kHz. A general linear increase was identified in the cancellation phase in both the ipsilateral and contralateral ear above 4 kHz. In the contralateral ear, the phase for cancellation reduced with increasing frequency in all three participants between 0.25 and 4 kHz. The test retest phase standard deviation was found to be greater in the contralateral cochlea when compared with ipsilateral and the standard deviation increased with higher frequencies. Suggesting small variations in BT position affect phase at the contralateral cochlea more than the ipsilateral cochlea.

There were significant variations in ipsilateral and contralateral levels needed for cancellation. These were both frequency and participant dependent and corresponded well with the existing literature (Pfiffner *et al*., 2011; Stenfelt, 2012), but are seen in much greater detail in our data. There were multiple large increases in inter-cochlear level differences of >10 dB identified over a relatively narrow frequency range (<0.5 kHz). Similarly, large negative inter-cochlear level differences were identified where the contralateral cochlea required 10 dB or more sound to cancel than the ipsilateral cochlea. The large increases and decreases over a narrow frequency range are thought to be due to resonance and anti-resonance (Stenfelt, 2012).

Future studies will focus on using the measured phase and level values in a cross-talk cancellation system
